# Supplementary material for: Wide field of view large aperture meta-doublet eyepiece
Source: Light Sci Appl. 2025 Jan 2;14:17. doi: 10.1038/s41377-024-01674-0 (PMC11693764; doi:10.1038/s41377-024-01674-0)
Supplement: Supplementary file 1 — Supplementary materials [file 41377_2024_1674_MOESM1_ESM.pdf]

Supplementary Information for Wide Field of View Large Aperture Meta-Doublet Eyepiece

Anna Wirth-Singh[1*], Johannes E. Fröch[1,2], Fan Yang[3], Louis Martin[3], Hanyu Zheng[3], Hualiang Zhang[4], Quentin T. Tanguy[2], Zhihao Zhou[2], Luocheng Huang[2], Demis D. John[5], Biljana Stamenic[5], Juejun Hu[3], Tian Gu[3], Arka Majumdar[1,2*]

[1]Department of Physics, University of Washington, Seattle, WA 98195, USA.
[2]Department of Electrical and Computer Engineering, University of Washington, Seattle, WA 98195, USA.
[3]Department of Materials Science and Engineering, Massachusetts Institute of Technology, Cambridge, MA 02139, USA.
[4]Department of Electrical and Computer Engineering, University of Massachusetts, Lowell, MA 01854, USA.
[5]Department of Electrical and Computer Engineering, University of California, Santa Barbara, CA 93106, USA.

*Corresponding author(s). E-mail(s): annaw77@uw.edu; arka@uw.edu;

S1 Meta-optics Design

The meta-optics were modeled using ray tracing software (Zemax OpticStudio), wherein the meta-optics were modeled as Binary-2 type surfaces. That is, the surfaces impart a phase delay given by the radially symmetric polynomial

$$\Phi(\rho) = \sum_{i=1}^{10} A_i (\frac{\rho}{M})^{2i} \tag{1}$$

where M is a normalization constant, ρ is the radial coordinate, and A_i are polynomial coefficients. The polynomial coefficients were fit to optimize the metasurface phase

profiles. In this case, we defined input fields at incident angles from 0° to 40° in 5° increments and weighted them equally during optimization. In addition, the air gap between the optical windows was also allowed to vary during optimization. The optimized coefficients A_i for both 1 cm and 2 cm designs are provided in Table S1.

Table S1 Metasurface Phase Coefficients

	1 cm MS1	1 cm MS2	2 cm MS1	2 cm MS2
M	220	4.9499019	880	880
A_1	-39030668	-5396.6394	-2.5301487e+08	-5065492.6
A_2	-3.4998936e+09	-4605.9085	3.1181838e+10	3.617131e+11
A_3	1.1770126e+14	52695.076	-1.5514954e+15	-1.4121207e+16
A_4	-1.6476049e+18	-170751.14	3.9689123e+19	5.699969e+20
A_5	1.2079497e+22	165018.88	-6.00699e+23	-5.589307e+24
A_6	-5.2122204e+25	273878.4	5.5958326e+27	-1.4182956e+29
A_7	1.3689181e+29	-6511.3499	-3.211279e+31	3.0013409e+33
A_8	-2.1507485e+32	-2438339	1.0918782e+35	2.2855181e+36
A_9	1.8577437e+35	4093040.3	-1.9629298e+38	-3.7326393e+41
A_{10}	-6.7821622e+37	-2029151.3	1.3505338e+41	2.058759e+45

To implement these phase profiles in a physical structure, we utilize the local phase approximation to map between the desired phase and pillar geometry. Under the local phase approximation, the unit cells are assumed to be locally periodic and more complex interactions between adjacent unit cells are ignored [1, 2]. These approximations may introduce small deviations in the wavefront from the Zemax design. In order to simulate these effects, full-wave simulations (e.g. FDTD) would be required, which is not practical for a metalens of this size. However, the agreement between simulation (via Zemax) and experiment attests to the fact that the local phase approximation is a very good approximation for meta-optics, at least focusing meta-optics. Our experimental results thus indicate that ray optics based simulators can be used to model meta-optics, which is significant because ray optics can be used to model large aperture optics.

In Figure S1a and S1b, we plot the scatterer unit cell phase and transmission as calculated by rigorous coupled wave analysis (RCWA) [3]. For ease of fabrication, all pillars are the same height and have varying square widths. The scatterers are 750 nm SiN square pillars on quartz substrate, arranged on a 350 nm periodicity lattice. The simulation wavelength is 633 nm. The calculated phase and amplitude values of the eleven selected unit cells are provided in Table S2. Because there is minimal variation in phase at increasing angle of incidence, we use the phase response at normal incidence to map from desired phase to pillar width. While the phase and amplitude responses are not optimal under certain off-axis angles, these imperfections mostly affect the focusing efficiency rather than the imaging quality of the metalenses. The phase errors are mostly random while the overall phase coverage is still approximately 2π. As a result, the phase errors, along with amplitude reduction of certain meta-atoms, contribute to background noise and reduce focusing efficiency.

To estimate the result of these imperfections on the optical performance, we performed diffraction modeling to simulate the PSFs of the 1 cm eyepiece design under

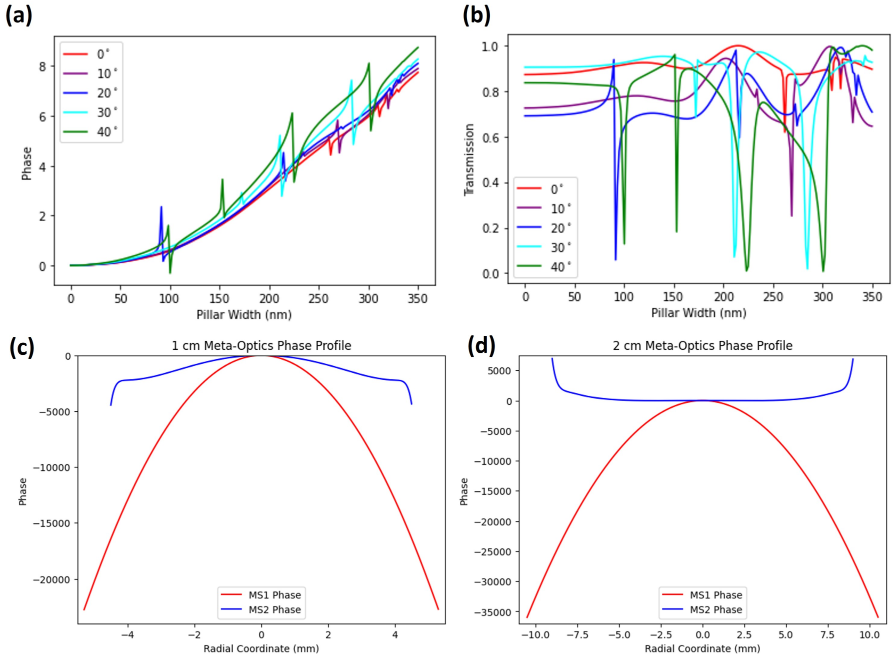

Fig. S1 Scatterer simulations and phase profiles. (a) The simulated scatterer unit cell phase as a function of pillar width, for increasing angles of incidence. (b) The simulated scatterer unit cell transmission as a function of angle of incidence. (c) The desired phase profiles for the 1 cm meta-optics. (d) The desired phase profiles for the 2 cm meta-optics.

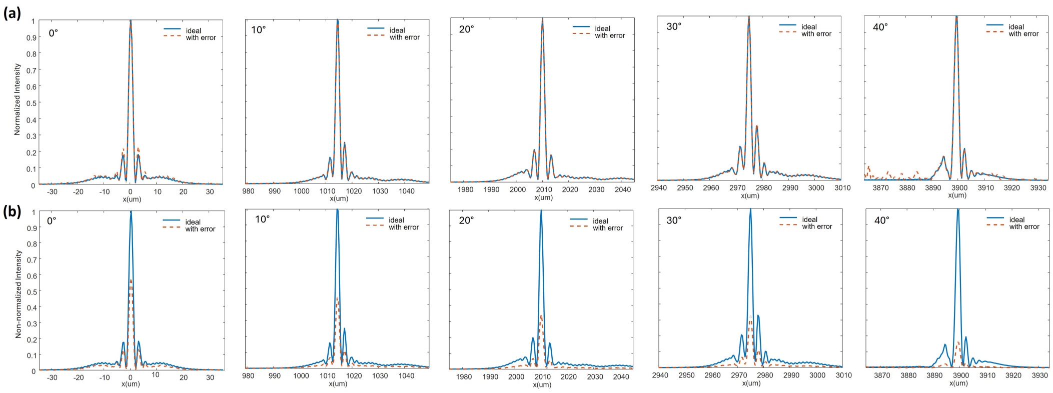

Fig. S2 Simulated PSFs considering the angle-dependent phase and amplitude responses of the meta-atoms for the 1 cm eyepiece optics. (a) The simulated PSFs in the ideal (blue) and with error (red) cases for angles of incidence from 0° up to 40°. Each PSF is normalized to its own maximum intensity. (b) The same as in (a), but normalized to a global maximum intensity. Decreased PSF intensity is observed at larger angles of incidence.

different angles of incidence using the angular spectrum method. The meta-optics are modeled as phase/amplitude masks using the angle-dependent simulated meta-atom responses in Table S2. Since 3D simulations for meta-optics of this size would be very computationally expensive, we performed 2D simulations (i.e., cylindrical lenses) instead and compared the results to the case assuming ideal phase/amplitude masks. These results are shown in Figure S2. After normalization of the peak intensity (Figure S2a), the PSFs taking angle-dependent phase and amplitude errors into account agree closely with the ideal case, indicating that the optical system can perform the imaging function as desired despite these errors. However, these errors do result in a reduction of peak intensity, as shown in the non-normalized results in Figure S2b.

In Fig. S1c and S1d, we plot the desired phase profiles for both metasurfaces (MS1 in red and MS2 in blue) for both the 1 cm and 2 cm optics. As described in the main text, MS1 performs the majority of the lensing function and is similar to a quadratic phase profile for large FoV [4]. The second metasurface acts as a corrector and entrance aperture and has a smaller phase gradient. In Fig. S3, we show the simulated aberration and distortion curves for both the 1 cm and 2 cm designs, respectively.

Table S2 Simulated meta-atom phase and amplitude for the selected pillar widths w.

	w (nm)	80	123	151	174	194	213	233	252	280	302	310
0°	Phase	0.00	0.63	1.25	1.88	2.51	3.13	3.78	4.37	5.03	5.67	6.28
	Amp	0.90	0.92	0.90	0.91	0.96	1.00	0.97	0.91	0.87	0.89	0.69
10°	Phase	0.00	0.66	1.28	1.91	2.60	3.29	3.98	4.47	5.02	5.74	6.08
	Amp	0.76	0.77	0.75	0.81	0.92	0.91	0.77	0.67	0.83	0.98	0.99
20°	Phase	0.00	0.57	1.23	1.89	2.56	3.49	3.96	4.56	5.22	5.82	6.11
	Amp	0.75	0.7	0.69	0.69	0.81	0.98	0.84	0.72	0.71	0.87	0.95
30°	Phase	0.00	0.70	1.38	1.98	2.87	2.37	4.10	4.86	6.26	6.02	6.33
	Amp	0.91	0.94	0.95	0.94	0.92	0.16	0.97	0.94	0.62	0.84	0.88
40°	Phase	0.00	0.68	1.64	2.24	3.13	4.21	4.01	5.17	6.15	4.64	6.32
	Amp	0.82	0.87	0.96	0.88	0.80	0.57	0.67	0.70	0.54	0.03	0.99

Due to sub-wavelength periodicity (350 nm) and large aperture (> 1 cm), the number of scatterers contained within each metasurface is large. Specifically, each of the 1 cm meta-optics contains on the order of 10^8 scatterers and each of the 2 cm meta-optics contains 10^9 scatterers at full aperture. Therefore, generating the GDS files for the meta-optics is a memory-consuming operation and a number of steps were taken to reduce computational burden. Firstly, the desired phase was discretized into 11 levels corresponding to 11 unique pillar geometries selected from the pillar width - phase response shown in Figure S1a. This discretization is expected to reduce the Strehl Ratio by only 4% [5] and allows for cell referencing which reduces the final GDS size. Secondly, the rotational symmetry of the optics were utilized. A radial slice of 1/8 of the full aperture was generated and then appropriately rotated and copied at the last step of GDS generation to produce the full circular aperture. With these considerations, the file size of each 1 cm meta-optic is around 4 Gb.

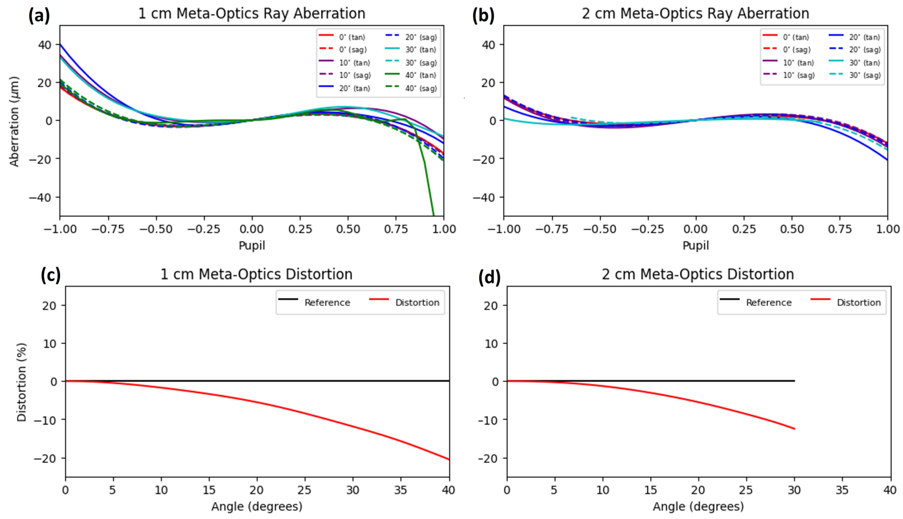

Fig. S3 Simulated aberration and distortion curves. (a,b) Ray aberration for the 1 cm (a) and 2 cm (b) meta-optics for the designed angles of indcidence, up to 40° for the 1 cm and up to 30° for the 2 cm. (c) Simulated distortion (%) for the 1 cm (c) and 2 cm (d) as a function of incident angle.

Fabrication constraints limit the writable area to approximately $0.80cm^2$ when using electron beam lithography, which is less than the area of the 2 cm meta-optics. To test the design, we fabricated only a slice of the 2 cm optics which were necessary to measure the point spread function (PSF). In those measurements, the 5.4 mm diameter pupil projects a beam of that size onto the meta-optics. As the angle of incidence is increased, that circular area is translated from the center of the meta-optics to the outer edge. Since the optic is radially symmetric, it is only necessary to characterize the PSF from angles of incidence in one direction. Therefore, we fabricated a slice of the optic which was 5.4 mm wide and extended 2.7 mm from the center in one direction (to fully cover the center area) to the full aperture (10.5 mm from center) in the other direction. Therefore, the total surface area of the fabricated slice was $0.71cm^2$. With rapidly improving nanolithography techniques, we anticipate that it will be possible in the near future to fabricate visible meta-optics with sub-100 nm resolution over a large surface area.

S2 Experiment Setup and Alignment Procedure

Detailed schematics of the experiment setup are shown in Figure S4. PSF measurements were done with the setup pictured in Fig. S4a and schematically depicted in S4b. First, fiber-coupled output from a HeNe laser (Newport N-LHP-131 at 632.8 nm wavelength) was collimated using a standard refractive lens. The collimated fiber output was mounted on a rotating arm which allows for incident angles up to approximately 45°. The iris, which serves as the pupil in this optical system, was placed directly above the axis of rotation. For ease of handling, the meta-optics substrates were attached to thin microscope slides and mounted 3-axis translation stages for

precise placement. The mounted meta-optics are shown in the inset of Fig. S4. The relay and imaging optics, which include a microscope objective (Nikon Plan Fluorite 20x, NA = 0.50, WD = 2.1 mm), tube lens, and the camera sensor (GT1930C), were mounted on a motorized translation stage to provide precise positioning along the z-axis. That stage, in turn, was mounted on a manual lateral stage to translate the entire collection system as necessary to collect PSFs at large FoV.

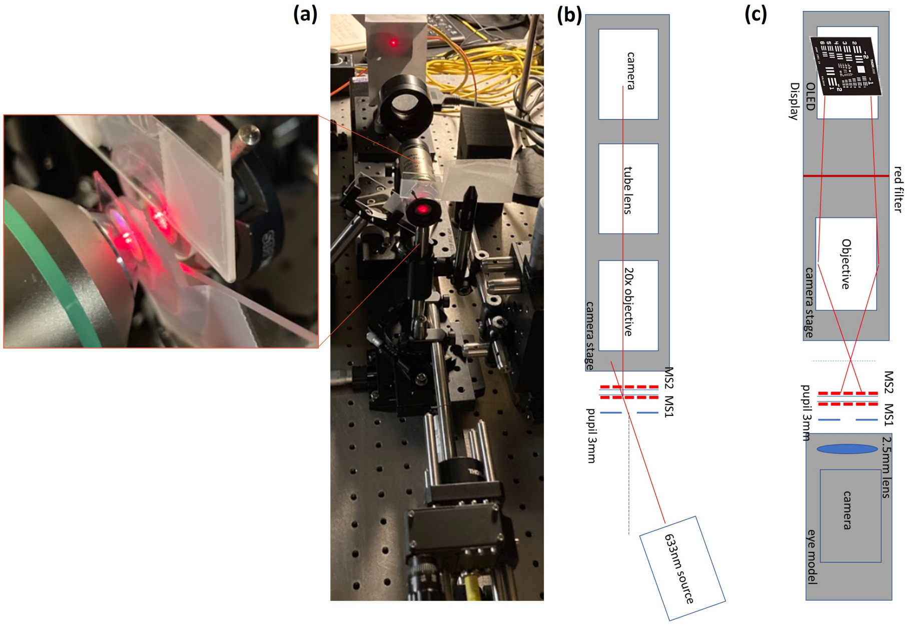

Fig. S4 Meta-optics characterization setup. (a) Photograph of the PSF measurement setup, with the inset (left) showing the mounted meta-optics and collecting objective. (b) Schematic of the PSF measurement setup. (c) Schematic of the imaging setup.

While the relay optics including the microscope objective are primarily used for magnifying the PSF, they have the additional function of aiding in the alignment of the meta-optic doublet system. By focusing the microscope objective on the surface of the meta-optic, the meta-optic surface can be precisely positioned. To successfully align the meta-optic doublet system, we first focused the microscope objective onto the surface of the iris. Using the motorized stage, we then moved the microscope objective backwards according to the designed spacing between the iris (pupil) and MS1. By then placing MS1 such that it is centered and focused in the microscope objective FoV, the metasurface has been correctly positioned. Repeating this procedure, we moved the microscope objective backwards according to the spacing between MS1 and MS2 and then place MS2 such that it is centered and in focus. Finally, by moving the camera stage backwards the desired focal plane, we are ready to measure the PSF of the aligned system.

The setup used for imaging experiments is schematically depicted in Figure S4c. In this case, a micro-LED displayed the desired image. A narrow linewidth red filter (Thorlabs FL632.8-1, 1 nm FWHM) was used to filter illumination to the design wavelength. Finally, the microscope objective was used to re-image the displayed image at the desired position. The width of the focused image was approximately 3 mm. The display, filter, and objective were mounted on a stage and translated laterally to produce images across the entire field of view. The meta-optics were placed according to the design and an iris was used as the pupil aperture. Finally, a refractive lens with 2.5 mm focal length focused the output on a camera, which composed the eye model.

S3 Full Aperture 2 cm Optics at Low Resolution with DUV Lithography

Electron beam lithography is one of the highest resolution nanofabrication techniques, capable of writing features only a few tens of nanometers in diameter. However, the technique is expensive and difficult to scale, making it unsuitable for commercial applications. To progress towards the goal of realizing large aperture meta-optics with commercially viable fabrication techniques, we designed and fabricated a full-aperture version of the 2 cm eyepiece doublet compatible with mass production-friendly deep ultraviolet (DUV) stepper lithography. While DUV is a rapidly improving technology, at present it cannot provide the same resolution as electron beam lithography.

The minimum feature size of our DUV lithography process is around 250 nm. Therefore, we adjusted the meta-optic unit cell to be compatible with the resolution limitations of DUV. For SiN-based visible meta-optics, the desired lattice periodicity is around $\lambda/2$ [6]; for the meta-optics presented in the main text, we use $\Lambda = 350$ nm, which requires a minimum feature (smallest pillar or gap) size around 80 nm to fully cover 0 to 2π phase range. For DUV lithography capable of producing 250 nm features, we identified a suitable set of scatterers with lattice periodicity of 1100 nm and pillar widths ranging from 300 nm to 600 nm. In addition, we increased the pillar height to 1750 nm to achieve the required 0 to 2π phase diversity. The phase and transmission responses of the 350 nm period and 1100 nm period scatterers are shown in Figure S6a and S6d, respectively. Due to the large periodicity relative to the wavelength in the 1100 nm case, the phase is not as well controlled. This results in an irregular phase response and reduced transmission as compared to the 350 nm case.

An additional negative consequence of the larger lattice periodicity is provides insufficient phase sampling to support the large phase gradient required of large FoV metasurfaces without introducing aliasing issues. While this does introduce undesirable effects, the meta-optics are still functional over a modest 40° full FoV. These issues and results are discussed below.

S3.1 DUV Fabrication

To demonstrate a path towards large aperture mass production of meta-optics, we selected DUV stepper lithography as a fabrication technique. The fabrication described herein was developed and carried out at the UCSB Nanofabrication Facility using an ASML 5500 DUV Stepper at 248 nm wavelength. The maximum die size was 21 mm

by 21 mm square. In this process, a chrome-on-glass mask is made at 4x magnification. In the stepper, DUV light is flooded through the mask and focused onto the sample. The mask is then ”stepped” over the surface of the wafer, in relatively quick exposures, to rapidly produce copies of the desired optic. With the initial investment of a high-quality mask, the subsequent copies can be made quickly and at a relatively low cost. In addition, to provide better etching uniformity over a larger area, we chose sapphire (n = 1.77) substrate due to its better thermal conductivity as compared to quartz for this large-aperture fabrication.

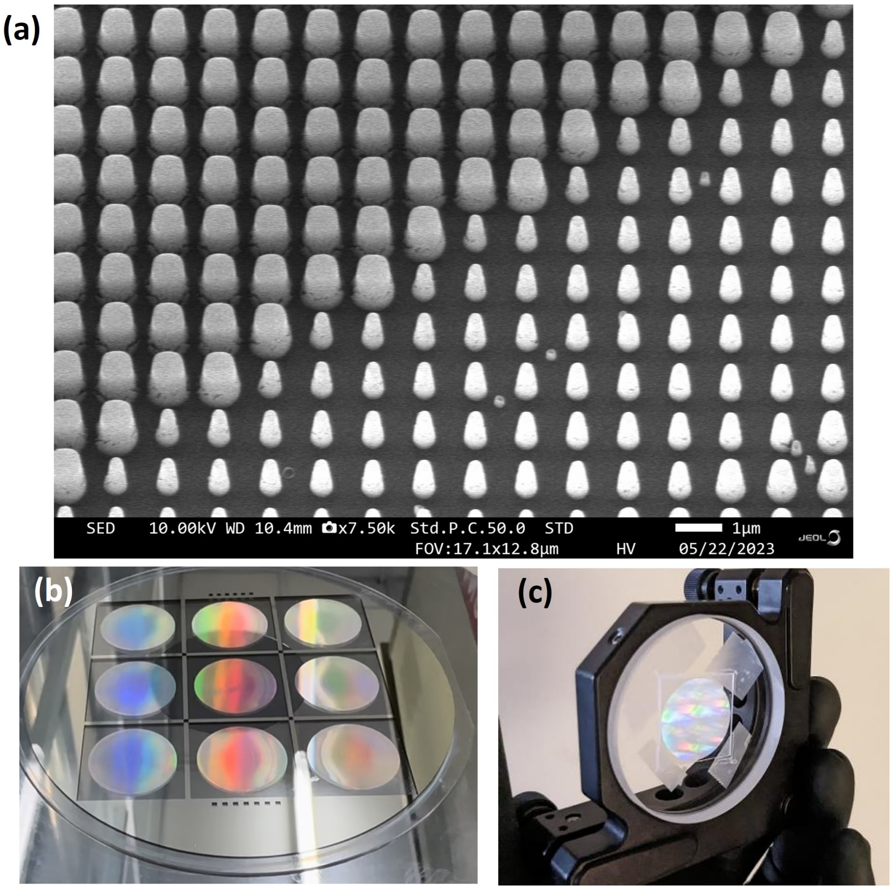

Fig. S5 Full 2 cm aperture meta-optics fabricated with DUV stepper lithography. (a) Scanning electron microscope image of the fabricated optics. (b) A wafer containing several fabricated meta-optics prior to dicing. (c) The 2 cm diameter MS2 mounted on BK7 spacer in a 2” mirror mount.

In detail, the sapphire substrates (650 μm thickness) were obtained from a commercial source and we deposited 1.75 μm SiN via PECVD at a temperature of 350C. Following this, a layer of Ruthenium (Ru) and silicon dioxide (SiO2) were deposited as a hard mask according to an established process [7]. Lithography was performed

using an ASML 5500 DUV Stepper with photomasks from Digidat, Inc. The SiO2 and Ru were subsequently etched to complete the hard mask. Next, the SiN was etched to the desired 1750 nm thickness. Finally, the SiO2 and Ru hardmasks were removed via dry etch. The fabricated wafers were diced to separate the individual meta-optics.

S3.2 Aliasing

Wide FoV meta-optics require a large phase gradient in order to modulate light at steep incident angles. An additional limitation on attainable FoV arises from the maximum phase gradient which can be supported by the meta-optics for a given lattice periodicity. If the required phase difference between adjacent meta-atoms becomes greater than π, aliasing effects occur. To avoid aliasing, the phase sampling of the meta-optic must satisfy the Nyquist-Shannon sampling theorem:

$$\Lambda < \frac{\pi}{|\Delta\phi(x,y)|_{max}} \tag{2}$$

where Λ is the sampling periodicity and $|\Delta\phi(x,y)|_{max}$ is the maximum absolute difference between adjacently sampled points in the spatial phase profile $\phi(x,y)$ [8]. To relate this to geometric optics, the generalized Snell's Law relates the phase gradient along the surface to the deflection angle:

$$n_1 sin(\theta_1) - n_0 sin(\theta_0) = \frac{\lambda}{2\pi}\frac{d\phi}{dx} \tag{3}$$

where n_1 and n_0 are the refractive indices of the input and output medium (in this case, for air, $n_1 = n_0 = 1$), θ_1 and θ_0 are the input and output deflection angles, λ is the incident wavelength, and $\frac{d\phi}{dx}$ is the phase gradient. From these two expressions, we can determine the maximum deflection angle of a metasurface for a given lattice periodicity:

$$\theta_{max} = sin^{-1}(\frac{\lambda}{2\Lambda}) \tag{4}$$

Equivalently, with numerical aperture (NA) defined as the sine of the maximum angle θ_{max} which can enter the optical system, we can directly relate the attainable NA to the wavelength and lattice periodicity:

$$NA \leq \frac{\lambda}{2\Lambda} \tag{5}$$

For lattice periodicity Λ which is smaller than half the wavelength ($\frac{\lambda}{2}$), angles up to nearly 90° can be supported. The maximum deflection angle supported by $\Lambda = 350$ nm is 65° (130° full FoV) for a single meta-optic. For the 1100 nm periodicity meta-optics, the maximum aliasing-free deflection angle is about 17° per optic. However, in a doublet system such as this one, this limitation can be mitigated by utilizing the additional degrees of freedom provided by the second surface. By stacking multiple metasurfaces, the overall deflection angle can be increased. We note a recent work [9] proposes several anti-aliasing strategies to circumvent this issue in metalenses, and

suggest that these strategies can also be employed to mitigate aliasing issues arising from insufficient phase sampling.

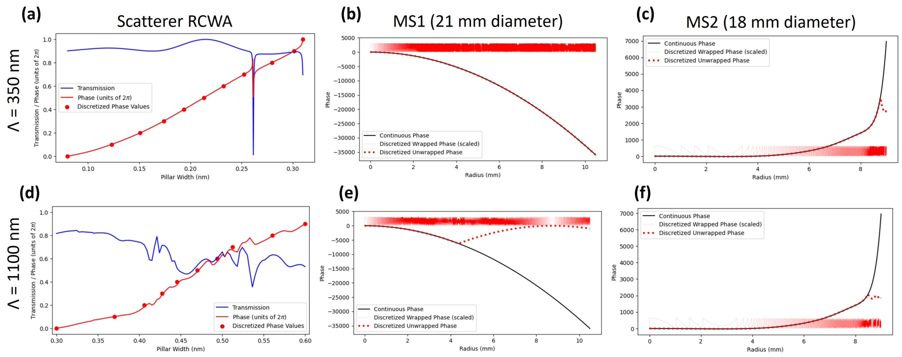

Fig. S6 High-resolution and low-resolution scatterer simulations and phase profiles. (a) The simulated high-resolution ($\Lambda = 350$ nm) scatterer unit cell phase and transmission versus pillar width. The SiN pillars are 750 nm tall. The dots indicate the discretized pillar values used in fabrication. (b) and (c) The desired (solid black line) and effective (dashed red line) phase profiles for the 2 cm meta-optics MS1 and MS2, respectively, using $\Lambda = 350$ nm. Since the periodicity is sufficiently small to support the desired phase gradient, the effective phase is the same as the desired phase. (d) The simulated low-resolution ($\Lambda = 1100$ nm) scatterer unit cell phase and transmission versus pillar width. The SiN pillars are 1750 nm tall. (e) and (f) The desired and effective phase profiles using $\Lambda = 1100$ nm. Aliasing effects are observed when the phase gradient becomes too large for the periodicity to support, indicated by the divergence of the desired phase profile (solid black line) and effective phase profile (dashed red line).

In Figure S6, we plot the desired and effective phase profiles for each 2 cm meta-optic for the high-resolution ($\Lambda = 350$ nm) and low-resolution ($\Lambda = 1100$ nm) scatterers. The desired continuous phase profiles for MS1 and MS2 are shown in black, the sampled phase profile modulo 2π is shown in red, and the effective phase profile given the phase sampling Λ is shown as a red dashed line. MS1, which accomplishes most of the lensing, has a phase gradient which increases approximately quadratically with the radial coordinate. The high-resolution scatterer periodicity provides sufficiently high phase sampling over the entire optic, so the effective phase gradient matches the desired phase gradient as shown in Fig. S6b. In MS2, the rapid increase in phase at the very edge is due to defining the optic diameter slightly larger than necessary; no rays interact with the outer edge, and as such the phase here is inconsequential to the performance of the system. For both MS1 and MS2, the lattice periodicity of 350 nm is sufficient to avoid any aliasing effects.

In contrast, the aliasing effect is significant in MS1 when sampling with 1100 nm lattice periodicity. In Figure S6(e), these effects are observed where the effective phase profile (red dashed line) diverges from the desired phase profile. Specifically, around $\rho = 4.2$mm, the desired phase difference between adjacently sampled points becomes greater than π. Near $\rho = 8.0$mm, the phase difference becomes 2π, resulting in an effective phase profile which is significantly different from the desired phase profile. Experimentally, this generates additional unwanted focal spots.

S3.3 Experiment Results

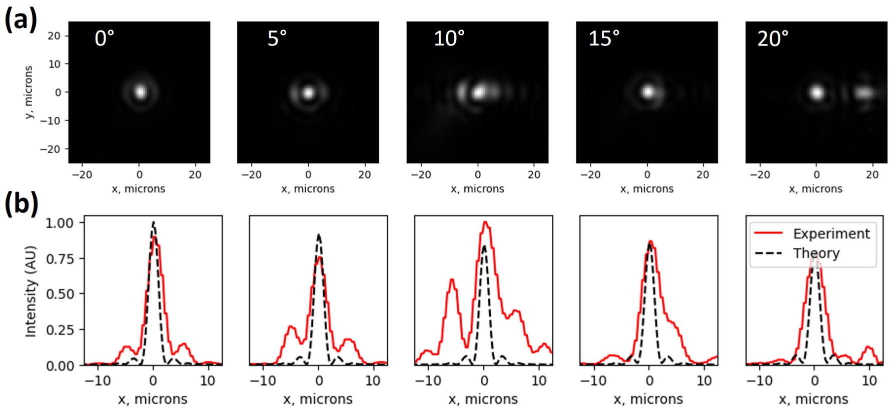

Fig. S7 PSF measurements of the full 2 cm aperture meta-optics fabricated using DUV lithography. (a) Experimental PSF measurements from 0° to 20° angle of incidence. (b) Horizontal line cuts of the PSFs shown in (a), with the experiment results shown as solid red lines and simulated results as dashed black lines. The simulated and experimental PSFs were normalized to their respective maximum intensity values.

Using the same experimental setup as for the other optics, we measured the PSF of the low-resolution 2 cm meta-optics up to an incident angle of 20° corresponding to 40° full FoV. These results are summarized in Figure S7. As expected, the performance is a bit worse than for the 2 cm meta-optic slice fabricated with electron beam lithography, but the PSFs are still relatively high-quality.

Supplementary References

[1] Zhao, M., Chen, M.K., Zhuang, Z.-P., Zhang, Y., Chen, A., Chen, Q., Liu, W., Wang, J., Chen, Z.-M., Wang, B., Liu, X., Yin, H., Xiao, S., Shi, L., Dong, J.-W., Zi, J., Tsai, D.P.: Phase characterisation of metalenses. Light: Science and Applications **10**(52) (2021)

[2] Zhelyeznyakov, M., Fröch, J., Wirth-Singh, A., Noh, J., Rho, J., Brunton, S., Majumdar, A.: Large area optimization of meta-lens via data-free machine learning. Communications Engineering **2**(60) (2023)

[3] Liu, V., Fan, S.: S4: A free electromagnetic solver for layered periodic structures. Computer Physics Communications (183), 2233–2244 (2012)

[4] Martins, A., Li, K., Li, J., Liang, H., Conteduca, D., Borges, B.-H.V., Krauss, T.F., Martins, E.R.: On metalenses with arbitrarily wide field of view. ACS Photonics **8**(7), 2073–2079 (2020)

[5] Aieta, F., Genevet, P., Kats, M., Capasso, F.: Aberrations of flat lenses and aplanatic metasurfaces. Optics Express **21**(25), 31530–31539 (2013)

[6] Zhan, A., Colburn, S., Trivedi, R., Fryett, T.K., Dodson, C.M., Majumdar, A.: Low-contrast dielectric metasurface optics. ACS Photonics **3**(2), 209–214 (2016)

[7] Mitchell, W.J., Thibeault, B.J., John, D.D., Reynolds, T.E.: Highly selective and vertical etch of silicon dioxide using ruthenium films as an etch mask. Journal of Vacuum Science and Technology A **4**(39), 043204 (2021)

[8] Zhan, A., Colburn, S., Dodson, C.M., Majumdar, A.: Metasurface freeform nanophotonics. Scientific Reports **7**(1673) (2017)

[9] Kim, S., Kim, J., Kim, K., Jeong, M., Rho, J.: Anti-aliased metasurfaces beyond the nyquist limit. arXiv Preprint (2024)
